# Checklist of British and Irish Hymenoptera - Proctotrupoidea

**DOI:** 10.3897/BDJ.4.e7936

**Published:** 2016-04-15

**Authors:** Gavin R. Broad

**Affiliations:** ‡ The Natural History Museum, London, United Kingdom

**Keywords:** Heloridae, Proctotrupidae, fauna, Ireland, Britain

## Abstract

**Background:**

A revised checklist of the British and Irish Heloridae and Proctotrupidae (Proctotrupoidea) substantially updates the previous comprehensive checklist, dating from 1978. Country level data (i.e. occurrence in England, Scotland, Wales, Ireland and the Isle of Man) is reported where known.

**New information:**

A total of three Heloridae and 39 Proctotrupidae (including only certainly recorded species) represents a 27% increase in the British list since 1978. Most species are still poorly known and there has been a dearth of taxonomic and faunistic work on the British and Irish fauna.

## Introduction

This paper is part of a series of new checklists to the British and Irish Hymenoptera fauna, that started with Broad and Livermore (2014b), Broad and Livermore (2014a) and Liston et al. (2014)and is the first update to the Hymenoptera checklist since 1978 (Fitton et al. 1978). Proctotrupoids are koinobiont endoparasitoids of holometabolous insect larvae. Although Proctrotrupidae can be common and readily collected by sweep netting or Malaise traps, the superfamily is poorly known biologically. Proctotrupidae are parasitoids of Coleoptera and, to a lesser extent, Diptera larvae, with one extraordinary host record from a centipede, whereas Heloridae have been reared from Neuroptera larvae (biology summarised in Gauld and Bolton 1988). Extralimital families attack Coleoptera and Neuroptera, or (Austroniidae, Peradeniidae, Proctorenyxidae) are completely unknown biologically.

The superfamily 'Proctotrupoidea' has generally been a welcoming home to a variety of parasitoid groups and recognised as not being monophyletic (Rasnitsyn 1988, Ronquist et al. 1999, Dowton and Austin 2001); in the 1978 checklist (Fitton et al. 1978) the Platygastroidea and Diaprioidea were included in the Proctotrupoidea. The removal of the Diapriidae, Maamingidae and Monomachidae to their own superfamily (Diaprioidea) has recently gained acceptance (e.g. Klopfstein et al. 2013). The Proctotrupoidea as presently defined (e.g. Sharkey 2007) now comprises the Heloridae and Proctotrupidae in Britain and the extralimital families Austroniidae, Pelecinidae, Peradeniidae, Proctorenyxidae, Roproniidae and Vanhorniidae.

**Figure 1.**
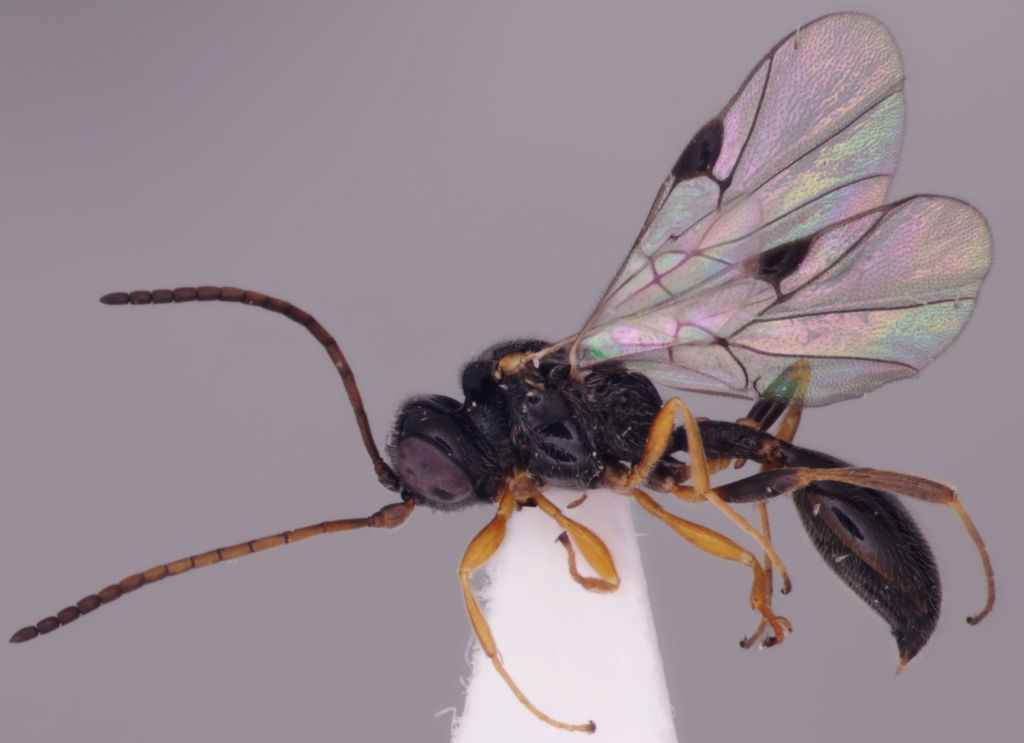
Representative British Heloridae. *Helorus ruficornis* Förster, female, England, Herts., Aldbury, 2013, coll. G.R. Broad.

**Figure 2.**
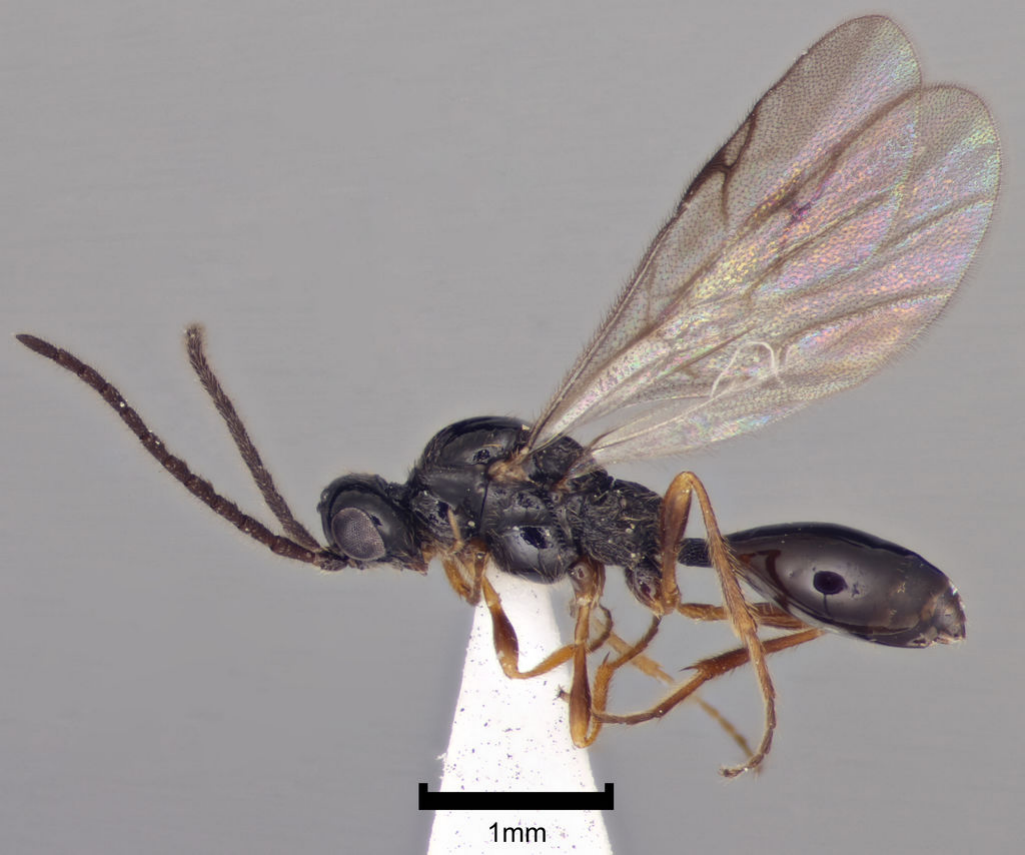
Representative British Proctotrupidae. *Disogmus basalis* (Thomson), male, England, Herefordshire, Mary Knoll Valley, 21-24.5.2007, coll. D.G. Notton, NHMUK010209578.

**Figure 3.**
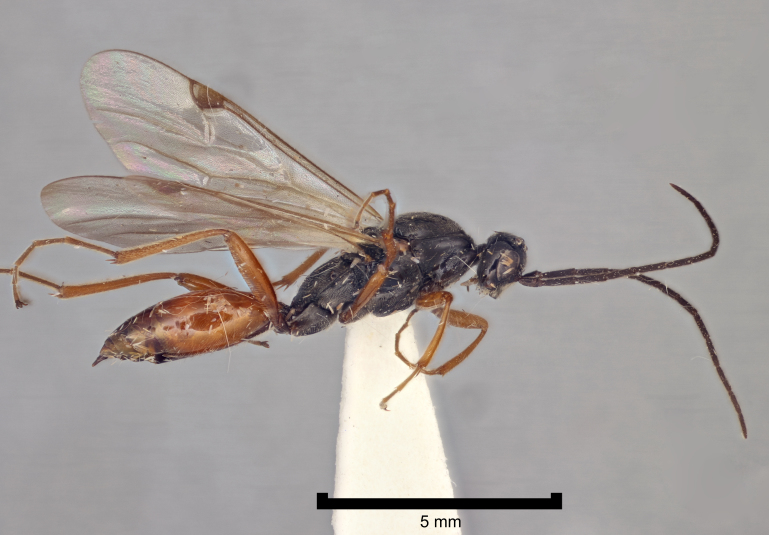
Representative British Proctotrupidae. *Proctotrupes brachypterus* (Schrank), female, England, Rothamsted, 26.9.1973, NHMUK010209579.

There has been very little work on the British proctotrupoid fauna and most of what we know about the British and Irish Proctotrupidae derives from the global monograph of Townes and Townes (1981). Both families can be recognised instantly by their distinctive wing venation and they each have a characteristic habitus (Figs 1, 2, 3). A revision of the British fauna would undoubtedly reveal more species.

## Materials and methods

Much of the distribution data are taken from Townes and Townes (1981). Distribution data pertaining to Ireland are mostly from O'Connor et al. (2004). Other sources are referred to. There is an excellent online resource, The Proctotrupidae of the World. The taxonomy follows Townes and Townes (1981) and Townes (1977) with subsequent changes referenced. We have tried to account for every name on the 1978 checklist Fitton et al. (1978) and all additions to the fauna since then have been referenced. A more complete methodology and rationale behind the checklist can be found in Broad (2014). The following conventions and abbreviations are used here:

[***species***] taxon deleted from the British and Irish list

NHM Natural History Museum, London

# known or suspected introductions with at least temporarily self-sustaining populations

? status (including uncertain synonymy) or identification in the British Isles uncertain

misident. has been misidentified as this name

nom. dub. *nomen dubium*, a name of unknown or doubtful application

nom. ob. *nomen oblitum*, ‘forgotten name’, does not have priority over a younger name

nom. nov. *nomen novum*, a replacement name

nom. nud. *nomen nudum*, an unavailable name, with no type specimen

preocc. name preoccupied (junior homonym)

stat. rev. *status revocatus*, revived status (e.g., raised from synonymy)

unavailable not meeting the requirements of the International Code of Zoological Nomenclature

var. variety, only available as a valid name under the provisions of article 45.6 of the ICZN

Alternative versions of the checklist, as a formatted Word document and Excel spreadsheet, are provided here in the supplementary materials: Suppl. materials 1, 2.

Photographs were taken using a Canon EOS 450D digital camera attached to a Leica MZ12 stereomicroscope and partially focused images were combined using Helicon Focus v.4.80 software.

## Checklists

### Proctotrupoidea

#### PROCTOTRUPOIDEA

#### Heloridae

Förster, 1856

##### Notes

Distribution data from Townes (1977), Fergusson and Smith (1974), Cooter and Fergusson (1993) and O'Connor et al. (2004).

#### Helorus

Latreille, 1802

COPELUS Provancher, 1881

#### Helorus anomalipes

(Panzer, 1798)

Sphex anomalipes Panzer, 1798
ater Latreille, 1802
paradoxus (Provancher, 1881, *Copelus*)
Helorus anomalipes ?*bifoveolata* Gregor, 1938
Helorus anomalipes ?*nigrotibia* Hellén, 1941

##### Distribution

England, Ireland

#### Helorus nigripes

Förster, 1856

rugosus Thomson, 1858

##### Distribution

England

#### Helorus ruficornis

Förster, 1856

coruscus Haliday, 1857
corruscus misspelling
flavipes Kieffer, 1907

##### Distribution

England, Ireland

#### Proctotrupidae

Latreille, 1802

##### Notes

Distribution data from Townes and Townes (1981) and O'Connor et al. (2004).

#### Brachyserphus

Hellén, 1941

#### Brachyserphus parvulus

(Nees, 1834)

Codrus parvulus Nees, 1834

##### Distribution

England, Ireland

#### Codrus

Panzer, 1801

#### Codrus niger

Panzer, 1805

##### Distribution

England, Ireland

#### Codrus picicornis

(Förster, 1856)

Disogmus picicornis Förster, 1856
subcompressus (Hedicke, 1927, *Phaenoserphus*)
vexator (Nixon, 1938, *Phaenoserphus*)
subclavatus (Hellén, 1941, *Phaenoserphus*)

##### Distribution

England, Ireland

##### Notes

Listed as *Phaenoserphus subcompressus* in Fergusson (1978).

#### Cryptoserphus

Kieffer, 1907

#### Cryptoserphus aculeator

(Haliday, 1839)

Proctotrupes aculeator Haliday, 1839
ater (Nees, 1834, *Codrus*) preocc.
perrisi (Kieffer, 1908, *Seprhus*)
deshii Drake, 1970, Cryptoserphus

##### Distribution

England, Scotland, Ireland

#### Cryptoserphus dilatus

Townes, 1981

##### Distribution

Ireland

##### Notes

added by Townes and Townes (1981)

#### Cryptoserphus flavipes

(Provancher, 1881)

Proctotrupes flavipes Provancher, 1881
brevimanus (Kieffer, 1908, *Serphus*)
longicalcar (Kieffer, 1908, *Serphus*)
cumaeus Nixon, 1938
fungorum Szelényi, 1940

##### Distribution

Ireland

#### Cryptoserphus longitarsis

(Thomson, 1858)

Proctotrupes longitarsis Thomson, 1858

##### Distribution

Ireland

#### Disogmus

Förster, 1856

#### Disogmus areolator

(Haliday, 1839)

Proctotrupes areolator Haliday, 1839
aequator Förster, 1856
discrepator Förster, 1856
elegans (Thomson, 1858, *Proctotrupes*)
nigripennis (Thomson, 1858, *Proctotrupes*)
canadensis Harrington, 1900
diversicornis Kieffer, 1906
glabratus Kieffer, 1906
carinatus Kieffer, 1907
torvus Whittaker, 1930

##### Distribution

England, Scotland, Ireland

#### Disogmus basalis

(Thomson, 1858)

Proctotrupes basalis Thomson, 1858
fuscitarsis Kieffer, 1907

##### Distribution

England, Scotland, Ireland

#### Exallonyx

Kieffer, 1904

##### Notes

unplaced species of *Exallonyx*:

[*Exallonyx leviventris* Kieffer, 1908 nom. dub., from England]

#### Exallonyx

Kieffer, 1904

#### Exallonyx (Exallonyx) ater

(Gravenhorst, 1807)

Codrus ater Gravenhorst, 1807
aterrimus (Dalla Torre, 1898, *Proctotrypes*)
xanthocerus Kieffer, 1908
filicornis Kieffer, 1908
syriacus Kieffer, 1908
gracilis Nixon, 1938

##### Distribution

England, Ireland

#### Exallonyx (Exallonyx) brevimala

Townes, 1981

##### Distribution

England, Ireland

##### Notes

added by Townes and Townes (1981)

#### Exallonyx (Exallonyx) confusus

Nixon, 1938

##### Distribution

England, Ireland

#### Exallonyx (Exallonyx) crenicornis

(Nees, 1834)

Codrus crenicornis Nees, 1834
clavipes (Thomson, 1858, *Proctotrupes*)
donisthorpei Kieffer, 1908
fumipennis Kieffer, 1908

##### Distribution

England, Scotland

##### Notes

Listed, as *Codrus fumipennis* (Kieffer), as doubtfully British by Fergusson (1978); status as a British species confirmed by Townes and Townes (1981).

#### Exallonyx (Exallonyx) formicarius

Kieffer, 1904

##### Distribution

England, Scotland, Ireland

#### Exallonyx (Exallonyx) ligatus

(Nees, 1834)

Codrus ligatus Nees, 1834

##### Distribution

England, Ireland

#### Exallonyx (Exallonyx) longicornis

(Nees, 1834)

Codrus longicornis Nees, 1834
micrurus (Keiffer, 1908, *Serphus*)

##### Distribution

Scotland, Ireland

#### Exallonyx (Exallonyx) microcerus

Kieffer, 1908

hyalinipennis Kieffer, 1908

##### Distribution

England, Ireland

##### Notes

Listed as a synonym of *Exallonyx formicarius* in Fergusson (1978).

#### Exallonyx (Exallonyx) minor

Townes, 1981

##### Distribution

England, Scotland, Ireland

#### Exallonyx (Exallonyx) nixoni

Townes, 1981

##### Distribution

England

#### Exallonyx (Exallonyx) pallidistigma

Morley, 1922

niger misident.
milleri (Tomšík, 1942, *Phaenoserphus*)

##### Distribution

England, Ireland

#### Exallonyx (Exallonyx) quadriceps

(Ashmead, 1893)

Proctotrypes quadriceps Ashmead, 1893
crassicornis Kieffer, 1908
hyalinipennis (Morley, 1922, *Proctotrypes*)

##### Distribution

England, Scotland, Ireland

##### Notes

Recorded by Townes and Townes (1981).

#### Exallonyx (Exallonyx) subserratus

Kieffer, 1908

curtigena Nixon, 1938

##### Distribution

England, Ireland

#### Exallonyx (Exallonyx) trichomus

Townes, 1981

##### Distribution

England, Ireland

##### Notes

added by Townes and Townes (1981)

#### Exallonyx (Exallonyx) trifoveatus

Kieffer, 1908

talpae Kieffer, 1908
borneanus (Cameron, 1912, *Proctotrypes*)
reicherti (Enderlein, 1916, *Proctotrupes*)
parvulus Brues, 1919
philonthiphagus Williams, 1932

##### Distribution

England, Ireland

##### Notes

added by Townes and Townes (1981)

#### Exallonyx (Exallonyx) wasmanni

Kieffer, 1904

myrmecophilus Kieffer, 1904
socialis Kieffer, 1908

##### Distribution

England, Ireland

##### Notes

Although excluded from the Irish list by O'Connor et al. (2004), the locality ‘Tanrego’ quoted by Townes and Townes (1981) refers to an estate in Sligo.

#### Eocodrus

Pschorn-Walcher, 1958

#### Exallonyx (Eocodrus) brevicornis

(Haliday, 1839)

Proctotrupes brevicornis Haliday, 1839
lineata Kieffer, 1908

##### Distribution

England, Scotland, Ireland

#### Mischoserphus

Townes, 1981

#### Mischoserphus arcuator

(Stelfox, 1950)

Cryptoserphus arcuator Stelfox, 1950
ione (Kozlov, 1971, *Cryptoserphus*)

##### Distribution

Ireland

#### Paracodrus

Kieffer, 1907

#### Paracodrus apterogynus

(Haliday, 1839)

Proctotrupes apterogynus Haliday, 1839
albipennis (Thomson, 1858, *Codrus*)
bethyliformis Kieffer, 1907

##### Distribution

Ireland

#### Parthenocodrus

Pschorn-Walcher, 1958

CRYPTOCODRUS Pschorn-Walcher, 1958

#### Parthenocodrus elongatus

(Haliday, 1839)

Proctotrupes elongatus Haliday, 1839
buccatus (Thomson, 1858, *Proctotrupes*)

##### Distribution

England, Ireland

#### Phaenoserphus

Kieffer, 1908

CARABIPHAGUS Morley, 1931

#### Phaenoserphus chittii

(Morley, 1922)

Proctotrypes chittii Morley, 1922
dubiosus Nixon, 1938

##### Distribution

England, Ireland

#### Phaenoserphus fuscipes

(Haliday, 1839)

Proctotrupes fuscipes Haliday, 1839

##### Distribution

Scotland, Ireland

#### Phaenoserphus gregori

Tomšík, 1942

##### Distribution

Ireland

#### Phaenoserphus pallipes

(Jurine, 1807)

Codrus pallipes Jurine, 1807
rufipes (Brullé, 1846, *Proctotrupes*)
testaceicornis (Kieffer, 1908, *Serphus*)

##### Distribution

England, Ireland

#### Phaenoserphus viator

(Haliday, 1839)

Proctotrupes viator Haliday, 1839
curtipennis (Haliday, 1839, *Proctotrupes*)
laevifrons (Förster, 1861, *Proctotrupes*)
sixianus (Vollenhoven, 1879, *Proctotrypes*)

##### Distribution

England, Ireland

#### Phaneroserphus

Pschorn-Walcher, 1958

##### Notes

species of *Phaneroserphus* removed from the British and Irish list:

[*cristatus* Townes, 1981] Fauna Europaea lists *Phaneroserphus cristatus* as occurring in Britain and France but Townes and Townes (1981) state that this species is only found in Japan, and no literature citations for its European occurrence can be traced.

#### Phaneroserphus calcar

(Haliday, 1839)

Proctotrupes calcar Haliday, 1839
calcaratus (Thomson, 1858, *Proctotrupes*)
seticornis (Thomson, 1858, *Proctotrupes*)
areolatus (Kieffer, 1908, *Serphus*)
castaneus (Kieffer, 1908, *Serphus*)

##### Distribution

England, Scotland, Ireland

#### Proctotrupes

Latreille, 1796

SERPHUS Schrank, 1780
ERODORUS Walckanaer, 1802
PROCTRUPES Rafinesque, 1815
PROCTOTRYPES Aggasiz, 1846
PROCTOTROPIS Gistel, 1848

#### Proctotrupes brachypterus

(Schrank, 1780)

Serphus brachypterus Schrank, 1780
divagator (Olivier, 1792, *Ichneumon*)
campanulator (Fabricius, 1798, *Ichneumon*)
emarciator (Fabricius, 1798, *Ichneumon*)
bimaculatus (Walckenaer, 1802, *Erodorus*)
brevipennis Latreille, 1802
bicolor Haliday, 1839
gladiator Haliday, 1839
sulcatus (Kieffer, 1908, *Serphus*)
hofferi (Tomšík, 1944, *Serphus*)
azarbajdzhanicus (Samedov, 1954, *Serphus*)

##### Distribution

England, Ireland

#### Proctotrupes gravidator

(Linnaeus, 1758)

Ichneumon gravidator Linnaeus, 1758
meridionalis Gribodo, 1880
rufigaster Provancher, 1881
collaris Szépligeti, 1901
suzukii Matsumura, 1912
zabriskiei (Brues, 1919, *Serphus*)

##### Distribution

England, Ireland

##### Notes

*Proctotrupes indivisus* (Kieffer, 1908, *Serphus*) removed from synonymy by Notton (2007).

#### Tretoserphus

Townes, 1981

##### Notes

species of *Tretoserphus* removed from the British and Irish list:

[*foveolatus* (Möller, 1882, *Proctotrupes*)] Listed as British by Fergusson (1978) as *perkinsi* was then considered to be a junior synonym of *foveolatus*.

#### Tretoserphus laricis

(Haliday, 1839)

Proctotrupes laricis Haliday, 1839
nigricauda (Kieffer, 1908, *Serphus*)
melanderi (Brues, 1919, *Cryptoserphus*)

##### Distribution

England, Ireland

#### Tretoserphus perkinsi

(Nixon, 1942)

Cryptoserphus perkinsi Nixon, 1942

##### Distribution

England, Ireland

## Supplementary Material

Supplementary material 1 Checklist of British and Irish Proctotrupoidea Data type: formatted text
Brief description: Word document version of the checklist
File: oo_74853.docx Broad, G.R.

Supplementary material 2 Checklist of British and Irish Proctotrupoidea Data type: spreadsheet
Brief description: Excel spreadsheet version of the checklist
File: oo_84638.xlsx Broad, G.R.

XML Treatment for PROCTOTRUPOIDEA

XML Treatment for Heloridae

XML Treatment for Helorus

XML Treatment for Helorus anomalipes

XML Treatment for Helorus nigripes

XML Treatment for Helorus ruficornis

XML Treatment for Proctotrupidae

XML Treatment for Brachyserphus

XML Treatment for Brachyserphus parvulus

XML Treatment for Codrus

XML Treatment for Codrus niger

XML Treatment for Codrus picicornis

XML Treatment for Cryptoserphus

XML Treatment for Cryptoserphus aculeator

XML Treatment for Cryptoserphus dilatus

XML Treatment for Cryptoserphus flavipes

XML Treatment for Cryptoserphus longitarsis

XML Treatment for Disogmus

XML Treatment for Disogmus areolator

XML Treatment for Disogmus basalis

XML Treatment for Exallonyx

XML Treatment for Exallonyx

XML Treatment for Exallonyx (Exallonyx) ater

XML Treatment for Exallonyx (Exallonyx) brevimala

XML Treatment for Exallonyx (Exallonyx) confusus

XML Treatment for Exallonyx (Exallonyx) crenicornis

XML Treatment for Exallonyx (Exallonyx) formicarius

XML Treatment for Exallonyx (Exallonyx) ligatus

XML Treatment for Exallonyx (Exallonyx) longicornis

XML Treatment for Exallonyx (Exallonyx) microcerus

XML Treatment for Exallonyx (Exallonyx) minor

XML Treatment for Exallonyx (Exallonyx) nixoni

XML Treatment for Exallonyx (Exallonyx) pallidistigma

XML Treatment for Exallonyx (Exallonyx) quadriceps

XML Treatment for Exallonyx (Exallonyx) subserratus

XML Treatment for Exallonyx (Exallonyx) trichomus

XML Treatment for Exallonyx (Exallonyx) trifoveatus

XML Treatment for Exallonyx (Exallonyx) wasmanni

XML Treatment for Eocodrus

XML Treatment for Exallonyx (Eocodrus) brevicornis

XML Treatment for Mischoserphus

XML Treatment for Mischoserphus arcuator

XML Treatment for Paracodrus

XML Treatment for Paracodrus apterogynus

XML Treatment for Parthenocodrus

XML Treatment for Parthenocodrus elongatus

XML Treatment for Phaenoserphus

XML Treatment for Phaenoserphus chittii

XML Treatment for Phaenoserphus fuscipes

XML Treatment for Phaenoserphus gregori

XML Treatment for Phaenoserphus pallipes

XML Treatment for Phaenoserphus viator

XML Treatment for Phaneroserphus

XML Treatment for Phaneroserphus calcar

XML Treatment for Proctotrupes

XML Treatment for Proctotrupes brachypterus

XML Treatment for Proctotrupes gravidator

XML Treatment for Tretoserphus

XML Treatment for Tretoserphus laricis

XML Treatment for Tretoserphus perkinsi
