# Supplementary material for: Checklist of British and Irish Hymenoptera - Proctotrupoidea
Source: Biodivers Data J. 2016 Apr 15;(4):e7936. doi: 10.3897/BDJ.4.e7936 (PMC4867700; doi:10.3897/BDJ.4.e7936)
Supplement: Supplementary material 1 — Checklist of British and Irish Proctotrupoidea [file biodiversity_data_journal-4-e7936-s001.docx]

Superfamily **Proctotrupoidea**

Gavin R. Broad

Recent phylogenetic studies suggest that the Proctotrupoidea should be broken up as it is not a monophyletic taxon (e.g. Rasnitsyn, 1988; Ronquist *et al.*, 1999; Dowton & Austin, 2001). The Proctotrupoidea as presently defined (e.g. Sharkey, 2007) now comprises the Heloridae and Proctotrupidae and the extralimital families Austroniidae, Pelecinidae, Peradeniidae, Proctorenyxidae, Roproniidae and Vanhorniidae.

Family **Heloridae** Förster, 1856^[[1]](#footnote-1)^

***Helorus*** Latreille, 1802

*COPELUS* Provancher, 1881

***anomalipes*** (Panzer, 1798, *Sphex*) E I

*ater* Latreille, 1802

*paradoxus* (Provancher, 1881, *Copelus*)

?*bifoveolata* Gregor, 1938

?*nigrotibia* Hellén, 1941

***nigripes*** Förster, 1856 E

*rugosus* Thomson, 1858

***ruficornis*** Förster, 1856 E I

*coruscus* Haliday, 1857

*corruscus* misspelling

*flavipes* Kieffer, 1907

Family **Proctotrupidae** Latreille, 1802^[[2]](#footnote-2)^

***Brachyserphus*** Hellén, 1941

***parvulus*** (Nees, 1834, *Codrus*) E I

***Codrus*** Panzer, 1801

***niger*** Panzer, 1805 E I

***picicornis*** (Förster, 1856, *Disogmus*)^[[3]](#footnote-3)^ E I

*subcompressus* (Hedicke, 1927, *Phaenoserphus*)

*vexator* (Nixon, 1938, *Phaenoserphus*)

*subclavatus* (Hellén, 1941, *Phaenoserphus*)

***Cryptoserphus*** Kieffer, 1907

***aculeator*** (Haliday, 1839, *Proctotrupes*) E S I

*ater* (Nees, 1834, *Codrus*) preocc.

*perrisi* (Kieffer, 1908, *Seprhus*)

*deshii* Drake, 1970, *Cryptoserphus*

***dilatus*** Townes, 1981 I added by Townes & Townes (1981)

***flavipes*** (Provancher, 1881, *Proctotrupes*) I

*brevimanus* (Kieffer, 1908, *Serphus*)

*longicalcar* (Kieffer, 1908, *Serphus*)

*cumaeus* Nixon, 1938

*fungorum* Szelényi, 1940

***longitarsis*** (Thomson, 1858, *Proctotrupes*) I

***Disogmus*** Förster, 1856

***areolator*** (Haliday, 1839, *Proctotrupes*) E S I

*aequator* Förster, 1856

*discrepator* Förster, 1856

*elegans* (Thomson, 1858, *Proctotrupes*)

*nigripennis* (Thomson, 1858, *Proctotrupes*)

*canadensis* Harrington, 1900

*diversicornis* Kieffer, 1906

*glabratus* Kieffer, 1906

*carinatus* Kieffer, 1907

*torvus* Whittaker, 1930

***basalis*** (Thomson, 1858, *Proctotrupes*) E S I

*fuscitarsis* Kieffer, 1907

***exallonyx*** Kieffer, 1904

Subgenus ***exallonyx*** Kieffer, 1904

***ater*** (Gravenhorst, 1807, *Codrus*) E I

*aterrimus* (Dalla Torre, 1898, *Proctotrypes*)

*xanthocerus* Kieffer, 1908

*filicornis* Kieffer, 1908

*syriacus* Kieffer, 1908

*gracilis* Nixon, 1938

***brevimala*** Townes, 1981 E I added by Townes & Townes (1981)

***confusus*** Nixon, 1938 E I

***crenicornis*** (Nees 1834, *Codrus*) E S^[[4]](#footnote-4)^

*clavipes* (Thomson, 1858, *Proctotrupes*)

*donisthorpei* Kieffer, 1908

*fumipennis* Kieffer, 1908

***formicarius*** Kieffer, 1904 E S I

***ligatus*** (Nees, 1834, *Codrus*) E I

***longicornis*** (Nees, 1834, *Codrus*) S I

*micrurus* (Keiffer, 1908, *Serphus*)

***microcerus*** Kieffer, 1908^[[5]](#footnote-5)^ E I

*hyalinipennis* Kieffer, 1908

***minor*** Townes, 1981 E S I

***nixoni*** Townes, 1981 E

***pallidistigma*** Morley, 1922 E I

*niger* misident.

*milleri* (Tomšík, 1942, *Phaenoserphus*)

***quadriceps*** (Ashmead, 1893, *Proctotrypes*) E S I^[[6]](#footnote-6)^

*crassicornis* Kieffer, 1908^[[7]](#footnote-7)^

*hyalinipennis* (Morley, 1922, *Proctotrypes*)^[[8]](#footnote-8)^

***subserratus*** Kieffer, 1908 E I

*curtigena* Nixon, 1938

***trichomus*** Townes, 1981 E I added by Townes & Townes (1981)

***trifoveatus*** Kieffer, 1908 E I added by Townes & Townes (1981)

*talpae* Kieffer, 1908

*borneanus* (Cameron, 1912, *Proctotrypes*)

*reicherti* (Enderlein, 1916, *Proctotrupes*)

*parvulus* Brues, 1919

*philonthiphagus* Williams, 1932

***wasmanni*** Kieffer, 1904 E I^[[9]](#footnote-9)^

*myrmecophilus* Kieffer, 1904

*socialis* Kieffer, 1908

Subgenus ***EOCODRUS*** Pschorn-Walcher, 1958

***brevicornis*** (Haliday, 1839, *Proctotrupes*) E S I

*lineata* Kieffer, 1908

Unplaced species of *Exallonyx*

[***leviventris*** Kieffer, 1908 nom. dub. E]

***mischoserphus*** Townes, 1981

***arcuator*** (Stelfox, 1950, *Cryptoserphus*) I

*ione* (Kozlov, 1971, *Cryptoserphus*)

***Paracodrus*** Kieffer, 1907

***apterogynus*** (Haliday, 1839, *Proctotrupes*) I

*albipennis* (Thomson, 1858, *Codrus*)

*bethyliformis* Kieffer, 1907

***Parthenocodrus*** Pschorn-Walcher, 1958

*CRYPTOCODRUS* Pschorn-Walcher, 1958

***elongatus*** (Haliday, 1839, *Proctotrupes*) E I

*buccatus* (Thomson, 1858, *Proctotrupes*)

***Phaenoserphus*** Kieffer, 1908

*CARABIPHAGUS* Morley, 1931

***chittii*** (Morley, 1922, *Proctotrypes*) E I

*dubiosus* Nixon, 1938

***fuscipes*** (Haliday, 1839, *Proctotrupes*) S I

***gregori*** Tomšík, 1942 I

***pallipes*** (Jurine, 1807, *Codrus*) E I

*rufipes* (Brullé, 1846, *Proctotrupes*)

*testaceicornis* (Kieffer, 1908, *Serphus*)

***viator*** (Haliday, 1839, *Proctotrupes*) E I

*curtipennis* (Haliday, 1839, *Proctotrupes*)

*laevifrons* (Förster, 1861, *Proctotrupes*)

*sixianus* (Vollenhoven, 1879, *Proctotrypes*)

***PHANEROSERPHUS*** Pschorn-Walcher, 1958

***calcar*** (Haliday, 1839, *Proctotrupes*) E S I

*calcaratus* (Thomson, 1858, *Proctotrupes*)

*seticornis* (Thomson, 1858, *Proctotrupes*)

*areolatus* (Kieffer, 1908, *Serphus*)

*castaneus* (Kieffer, 1908, *Serphus*)

[***cristatus*** Townes, 1981^[[10]](#footnote-10)^]

***Proctotrupes*** Latreille, 1796

*SERPHUS* Schrank, 1780 suppressed

*ERODORUS* Walckanaer, 1802

*PROCTRUPES* Rafinesque, 1815

*PROCTOTRYPES* Aggasiz, 1846

*PROCTOTROPIS* Gistel, 1848

***brachypterus*** (Schrank, 1780, *Serphus*) E I

*divagator* (Olivier, 1792, *Ichneumon*)

*campanulator* (Fabricius, 1798, *Ichneumon*)

*emarciator* (Fabricius, 1798, *Ichneumon*)

*bimaculatus* (Walckenaer, 1802, *Erodorus*)

*brevipennis* Latreille, 1802

*bicolor* Haliday, 1839

*gladiator* Haliday, 1839

*sulcatus* (Kieffer, 1908, *Serphus*)

*hofferi* (Tomšík, 1944, *Serphus*)

*azarbajdzhanicus* (Samedov, 1954, *Serphus*)

***gravidator*** (Linnaeus, 1758, *Ichneumon*)^[[11]](#footnote-11)^ E I

*meridionalis* Gribodo, 1880

*rufigaster* Provancher, 1881

*collaris* Szépligeti, 1901

*suzukii* Matsumura, 1912

*zabriskiei* (Brues, 1919, *Serphus*)

***TRETOSERPHUS*** Townes, 1981

***laricis*** (Haliday, 1839, *Proctotrupes*) E I

*nigricauda* (Kieffer, 1908, *Serphus*)

*melanderi* (Brues, 1919, *Cryptoserphus*)

***perkinsi*** (Nixon, 1942, *Cryptoserphus*) E I

species excluded from the British and Irish list

[***foveolatus*** (Möller, 1882, *Proctotrupes*)^[[12]](#footnote-12)^]

**References**

Cooter, J. & Fergusson, N. D. M. 1993. *Helorus nigripes* Förster (Hym., Heloridae), a fifth British record. *Entomologist's Monthly Magazine* **129**: 29.

Dowton, M. & Austin, A. D. 2001. Simultaneous analysis of 16S, 28S, COI and morphology in the Hymenoptera: Apocrita - evolutionary transitions among parasitic wasps. *Biological Journal of the Linnean Society* **74**: 87-111.

Fergusson, N. D. M. & Smith, K. G. V. 1974[1973]. *Helorus rugosus* Thomson (Hym., Heloridae) in Britain. *Entomologist's Monthly Magazine* **109**: 222.

Fitton, M. G., Graham, M. W. R. de V., Bouček, Z. R. J. Fergusson, N. D. M., Huddleston, T., Quinlan, J. & Richards, O. W. 1978. Kloet and Hincks. A check list of British insects. Part 4: Hymenoptera. *Handbooks for the Identification of British Insects* **11(4)**: ix + 159 pp.

Notton, D. G. 2007. A catalogue of types of the smaller taxa of Proctotrupoidea (Hymenoptera) in the Muséum national d’Histoire naturelle, Paris, with notes on the history of the insect collection of L. A. G. Bosc d’Antic. *Zoosystema* **29**: 457-470.

O'Connor, J. P., Nash, R., Notton, D. G. & Fergusson, N. D. M. 2004. A catalogue of the Irish Platygastroidea and Proctotrupoidea (Hymenoptera). *Occasional Publication of the Irish Biogeographical Society* **7**: 1-110.

Rasnitsyn, A. P. 1988. An outline of the evolution of the hymenopterous insects (order Vespida). *Oriental Insects* **22**: 115-145.

Ronquist, F., Rasnitsyn, A. P., Roy, A., Eriksson, E. & Lindgren, M. 1999. Phylogeny of the Hymenoptera: A cladistic reanalysis of Rasnitsyn's (1988) data. *Zoologica Scripta* **28**: 13-50.

Townes, H. 1977. A revision of the Heloridae (Hymenoptera). *Contributions of the American Entomological Institute* **15(2)**: 1-12.

Townes, H. K. & Townes, M. 1981. A revision of the Serphidae (Hymenoptera). *Memoirs of the American Entomological Institute* **32**: i-iv, 1-541.

1. Distribution data from Townes (1977), Fergusson & Smith (1974), Cooter & Fergusson (1993) and O’Connor *et al.* (2004). [↑](#footnote-ref-1)
2. Distribution data from Townes & Townes (1981) and O’Connor *et al.* (2004). [↑](#footnote-ref-2)
3. Listed as *Phaenoserphus subcompressus* in Fergusson (1978). [↑](#footnote-ref-3)
4. Listed, as *Codrus fumipennis* (Kieffer), as doubtfully British by Fergusson (1978); status as a British species confirmed by Townes & Townes (1981). [↑](#footnote-ref-4)
5. Listed as a synonym of *formicarius* in Fergusson (1978). [↑](#footnote-ref-5)
6. Recorded by Townes & Townes (1981). [↑](#footnote-ref-6)
7. Listed as a queried synonym of *ater* in Fergusson (1978). [↑](#footnote-ref-7)
8. Listed as a synonym of *ligatus* in Fergusson (1978). [↑](#footnote-ref-8)
9. Although excluded from the Irish list by O’Connor *et al.* (2004), the locality ‘Tanrego’ quoted by Townes & Townes (1981) refers to an estate in Sligo. [↑](#footnote-ref-9)
10. Fauna Europaea lists *Phaneroserphus cristatus* as occurring in Britain and France but Townes & Townes (1981) state that this species is only found in Japan, and no literature citations for its European occurrence can be traced. [↑](#footnote-ref-10)
11. *Proctotrupes indivisus* (Kieffer, 1908, *Serphus*) removed from synonymy by Notton (2007). [↑](#footnote-ref-11)
12. Listed as British by Fergusson (1978) as *perkinsi* was then considered to be a junior synonym of *foveolatus*. [↑](#footnote-ref-12)
